# Resting-state neural activity and connectivity associated with subjective happiness

**DOI:** 10.1038/s41598-019-48510-9

**Published:** 2019-08-20

**Authors:** Wataru Sato, Takanori Kochiyama, Shota Uono, Reiko Sawada, Yasutaka Kubota, Sayaka Yoshimura, Motomi Toichi

**Affiliations:** 1 Kokoro Research Center, Kyoto University, Kyoto University, 46 Shimoadachi, Sakyo, Kyoto, 606-8501 Japan; 2 Brain Activity Imaging Center, ATR-Promotions, 2-2-2 Hikaridai, Seika-cho, Soraku-gun, Kyoto, 619-0288 Japan; 3 Department of Neurodevelopmental Psychiatry, Habilitation and Rehabilitation, Graduate School of Medicine, Kyoto University, 53 Shogoin-Kawaharacho, Sakyo, Kyoto, 606-8507 Japan; 4 Health and Medical Services Center, Shiga University, 1-1-1, Baba, Hikone, Shiga, 522-8522 Japan; 5 Faculty of Human Health Science, Graduate School of Medicine, Kyoto University, 53 Shogoin-Kawaharacho, Sakyo-ku, Kyoto, 606-8507 Japan; 6 The Organization for Promoting Neurodevelopmental Disorder Research, 40 Shogoin-Sannocho, Sakyo, Kyoto, 606-8392 Japan

**Keywords:** Consciousness, Limbic system

## Abstract

The majority of people throughout the world rate subjective happiness as the top of the important thing in life. A recent structural neuroimaging study exploring neurocognitive mechanisms underlying subjective happiness has suggested that the gray matter volume of the right precuneus is associated with Subjective Happiness Scale (SHS) scores. However, how the neural activity in this region, as well as the neural functional coupling between this and other regions, could be related to SHS scores remains unclear. To investigate these issues, we performed resting-state functional magnetic resonance imaging and analyzed the fractional amplitude of low-frequency fluctuation (fALFF) in participants, whose subjective happiness was evaluated using the SHS. Lower fALFF values in the right precuneus were associated with higher SHS scores. Furthermore, functional connectivity and spectral dynamic causal modeling analyses showed that both functional and effective connectivity of the right precuneus with the right amygdala were positively associated with SHS scores. These findings, together with other evidence on the information-processing functions of these brain regions, suggest the possibility that subjective happiness is associated with a reduction in self-referential mental processes, which are well integrated with emotional processing.

## Introduction

International surveys have found that happiness is rated as the top of the most important thing in life by the majority of people throughout the world^1^. To measure subjective happiness adequately, a previous psychological study developed the Subjective Happiness Scale (SHS)^2^. While other scales measuring subjective happiness are either single-item global evaluations (and hence not conducive to testing psychometric properties) or assess only selected (i.e., emotional or cognitive) components of subjective happiness, the SHS is a four-item measure of global subjective happiness^2^. The SHS assesses subjective happiness at a global level^2^ or characteristic level^3^ as “more enduring than momentary or daily happiness but that is also somewhat malleable over time”^3^. SHS scores are not affected by short-term (e.g., a few months) pleasure^4^. A number of previous studies have used the SHS and have shown its good-to-excellent reliability and validity with diverse populations^5^. These data suggest that the SHS may be an appropriate instrument with which to measure subjective happiness.

To explore the neural mechanisms underlying subjective happiness, a recent structural neuroimaging study has investigated the gray matter volume in association with SHS scores^6^. The researchers found that the volume of the right precuneus was associated with SHS scores. The results indicate that subjective happiness can be measured with objective biomarkers. Furthermore, the results suggest information-processing mechanisms underlying subjective happiness based on the findings regarding the information-processing functions of the precuneus^7^.

However, what type of precuneus activity (i.e., hyper- or hypo-activity) could be related to SHS scores remains unknown. This information would be valuable to further specify the functional association between subjective happiness and the precuneus. To assess brain activity related to stable psychological constructs such as subjective happiness, several functional magnetic resonance imaging (fMRI) studies have indicated that low-frequency (<0.1 Hz) resting-state neural activity in the absence of tasks may be a suitable measure^8,9^. The fractional amplitude of low-frequency fluctuation (fALFF) during the resting state has been proposed to reflect the intensity of spontaneous neural activity^10,11^. Although, to the best of our knowledge, the association between resting-state brain activation and SHS scores has not been investigated to date, there are some clues regarding this issue. First, trainees of mindfulness meditation^12^, which reportedly heightens SHS scores^13^ and increases the gray matter volume in the precuneus^14^, showed reduced precuneus activity when resting compared with normal participants^15^. Second, in patients with depression, who exhibit low SHS scores^16^ and a reduced gray matter volume in the precuneus^17,18^, resting-state blood flow in the precuneus was increased during depressive episodes^19,20^ and was decreased after improvement of their mental health^21^. Based on these data, we hypothesized that fALFF values in the precuneus would be negatively associated with SHS scores.

Furthermore, functional coupling between the precuneus and other brain regions associated with SHS scores remains unknown. Regarding functional coupling during the resting state, several previous fMRI studies have shown that resting-state functional connectivity, i.e., the correlation among spontaneous slow fluctuations in the hemodynamic signals of brain regions, can indicate the functional neural system status^8,22,23^. Correlations and anti-correlations among brain regions have been proposed to integrate and segregate neuronal activity, respectively, in the functional neural system^24^. Because subjective happiness is a composite of emotional and cognitive elements^2^ and the precuneus receives projections from widespread cortical and subcortical regions^25^, the precuneus may integrate information from other brain regions to implement subjective happiness^6^. The following considerations suggest that the amygdala may be one of the brain regions that send emotional information to the precuneus: (1) ample neuroscientific evidence has indicated that the amygdala is involved in the emotional processing of environmental stimuli, such as evaluations of the emotional significance of stimuli^26,27^; (2) anatomical studies in monkeys showed that the amygdala sends direct projections to the medial parietal region^25,28^; and (3) several previous fMRI studies found reduced resting-state functional connectivity between the precuneus and amygdala in depressed patients^29–31^. Based on these data, we hypothesized that higher SHS scores would be associated with stronger positive functional connectivity between the precuneus and amygdala.

In addition, the direction of connectivity between the precuneus and amygdala may be relevant. In contrast to functional connectivity, which is inherently non-directional, a dynamic causal modeling (DCM) approach suggests directional effective connectivity. Although originally developed as an analytic tool for task-related fMRI data^32^, DCM was recently extended, as spectral DCM (spDCM), to accommodate resting-state analysis^33,34^. Based on the above psychological and neural models, we hypothesized that the information sent via effective connectivity from the amygdala to the precuneus would be reflected in SHS scores.

To investigate these hypotheses, we analyzed resting-state functional MRI and SHS scores^2^. First, as the index of spontaneous neural activity during the resting state, we analyzed fALFF. Then, functional connectivity analyses and spDCM were conducted to assess functional coupling between brain regions.

## Results

### fALFF associated with SHS scores

**Figure 1:**
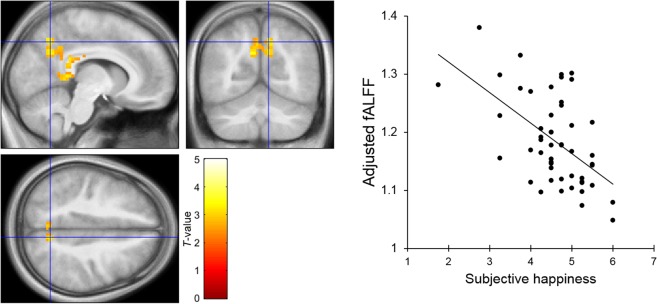
Brain regions showing a significant negative association between the subjective happiness score and fractional amplitude of low-frequency fluctuation (fALFF) value. (Left) A statistical parametric map (*p* < 0.05, cluster-level family-wise error corrected) for the group analysis of the fALFF map. The area is overlaid on the mean spatially normalized structural magnetic resonance images. The blue cross indicates the location of the peak voxel (*x* = 3, *y* = −72, *z* = 36). The red–white color scale indicates the *T*-values. (Right) A scatter plot of the adjusted fALFF values as a function of the subjective happiness score at the peak voxel. Effects of no interest (age, sex, full-scale intelligence quotient, and mean framewise displacement) were regressed out.

To identify the spontaneous brain activity associated with SHS scores, fALFF was analyzed using a voxel-wise multiple regression analysis with family-wise error- (FWE-) correction for the whole brain, treating SHS scores as the independent variable and sex, age, full-scale intelligence quotient (IQ), and framewise displacement (FD)^35^ as covariates. The results showed a significant negative relationship between SHS scores and fALFF values in the right precuneus (Table 1; Fig. 1). To scrutinize the results of the voxel-wise analysis, we further conducted region of interest (ROI) analyses. The partial correlation coefficient (controlling for sex, age, full-scale IQ, and FD effects) between the fALFF value at the peak voxel (*x* = 3, *y* = −72, *z* = 36) and the SHS scores was significant (*r* = −0.52, *p* < 0.001); the results remained unchanged when the first eigenvariate of the fALFF values over the whole cluster (70 voxels) was used (*r* = −0.64, *p* < 0.001) (Supplementary Fig. 1). The voxel-wise multiple regression analysis showed that the SHS scores were not significantly associated with fALFF values in any other brain region.

**Table 1:** Brain regions that exhibited a significant negative association with the subjective happiness score (*p* < 0.05, cluster-level corrected).

Brain region	BA	Coordinates			*Z*-value	Cluster size (voxels)
		*x*	*y*	*z*		
R. Precuneus	7	3	−72	36	3.74	70
L/R. Precuneus	7	0	−69	45	3.66	
R. Superior parietal lobule	7	21	−75	57	3.73	59
R. Precuneus	7	9	−72	57	2.75	

BA, Brodmann’s area; L, left: R, right.

### Functional connectivity associated with SHS scores

**Figure 2:**
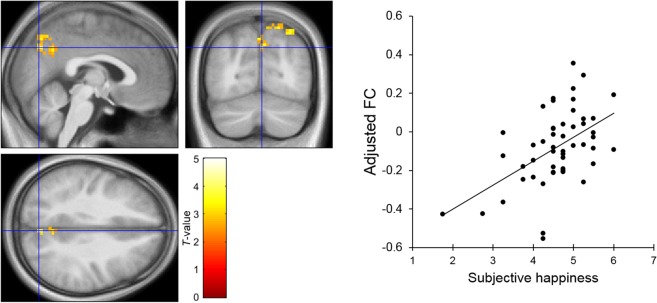
Brain regions showing a significant positive association between the subjective happiness score and functional connectivity (FC) values seeded from the right amygdala. (Upper) A statistical parametric map (*p* < 0.05, cluster-level family-wise error corrected) for the group analysis of the seed-based functional connectivity map. The area is overlaid on the mean spatially normalized structural magnetic resonance images. The blue cross indicates the location of the peak voxel (*x* = 9, *y* = −60, *z* = 42). The red–yellow color scale indicates the *T*-values. (Right) A scatter plot of adjusted functional connectivity parameters as a function of the subjective happiness score at the peak voxel. Effects of no interest (age, sex, full-scale intelligence quotient, and mean framewise displacement) were regressed out.

To identify the spontaneous functional coupling between brain regions associated with SHS scores, functional connectivity was analyzed. Based on our hypothesis regarding the amygdala, as described in the Introduction, activity in the left and right amygdala was used as the seed signals, and the functional connectivity associated with SHS scores was examined. Voxel-wise multiple regression analyses were conducted in a similar manner as the above analysis. The results showed that SHS scores were significantly and positively associated with functional connectivity between the right amygdala and right medial parietal regions, including the precuneus (Table 2; Fig. 2). The ROI-based partial correlation coefficient (controlling for sex, age, full-scale IQ, and FD effects) between functional connectivity at the peak voxel (*x* = 9, *y* = −60, *z* = 42) and the SHS scores was significant (*r* = 0.48, *p* < 0.001); the results remained unchanged when the first eigenvariate of the connectivity values over the whole cluster (432 voxels) was used (*r* = 0.57, *p* < 0.001) (Supplementary Fig. 1). The voxel-wise analyses showed that no other functional connectivity was significantly associated with SHS scores.

**Table 2:** Brain regions that exhibited significant functional connectivity with the right amygdala positively associated with the subjective happiness score (*p* < 0.05, cluster-level corrected).

Brain region	BA	Coordinates			*Z*-value	Cluster size (voxels)
		*x*	*y*	*z*		
R. posterior cingulate cortex	29	12	−42	18	4.35	432
R. Precuneus	7	9	−60	42	3.66	

BA, Brodmann’s area; R, right.

To explore other functional connections associated with SHS scores, we conducted functional connectivity analysis using the precuneus foci activity detected in the above fALFF analysis as the seed signals. The results confirmed that, although it did not reach our predefined level of significance, the functional connectivity of a small cluster in the right amygdala was positively associated with SHS scores (*x* = 24, *y* = −3, *z* = −15; *t*(45) = 2.57, *k* = 2). The functional connectivity of other regions with the precuneus was not significantly associated with SHS scores.

### Effective connectivity associated with SHS scores

To clarify the direction of functional coupling between the amygdala and precuneus associated with SHS scores, spDCM was performed. First, we constructed four hypothesized network models for each participant. The four models had either bidirectional, unidirectional, or no extrinsic (between-region) connections between the amygdala and precuneus. We assumed that both the amygdala and precuneus had inhibitory self (recurrent) connections by default. We selected the optimal network structure using random-effects Bayesian model selection (BMS)^36^. The exceedance probability of the random-effects BMS indicated that the model with bidirectional connectivity between the amygdala and precuneus was the most likely (Supplementary Fig. 2).

**Figure 3:**
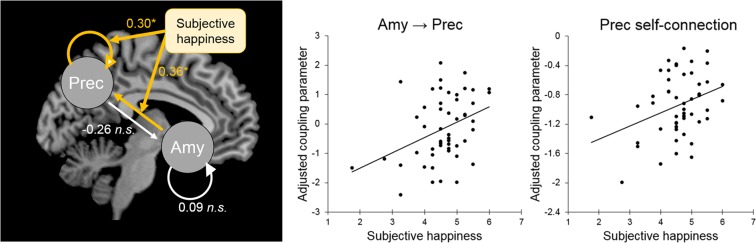
Significant effective connectivity related to the subjective happiness score. (Left) Models analyzed by spectral dynamic causal modeling. Arrows indicate extrinsic (between-region) and inhibitory self (recurrent) connections in the amygdala (Amy) and precuneus (Prec). Yellow and white lines indicate the locations of the coupling parameters significantly and non-significantly associated with subjective happiness scores, respectively. Values represent standardized coupling parameters indicating the associations with subjective happiness scores. Note that positive intra-regional self-connectivity indicates inhibitory effects. (Right) Scatter plot of the adjusted coupling parameters of amygdala → precuneus connectivity and precuneus self-connectivity as functions of the subjective happiness scores. Effects of no interest (age, sex, full-scale intelligence quotient, and mean framewise displacement) were regressed out.

Next, we assessed associations between coupling parameters and SHS scores. The coupling parameters of the bidirectional model were estimated by fitting the observed cross-spectral data to the model using variational Bayesian techniques. Multiple regression analyses were then conducted using the coupling parameters as the dependent variables, SHS scores as the independent variable, and sex, age, full-scale IQ, and FD as covariates of no interest. Among inter-regional coupling parameters, the connectivity from the amygdala to the precuneus was significantly and positively associated with SHS scores (*β* = 0.36, *t*(45) = 2.51, *p* < 0.05, *r* = 0.35; Fig. 3); however, this was not the case for the connectivity from the precuneus to the amygdala (*β* = −0.26, *t*(45) = 1.71, *p* = 0.09, *r* = −0.25). Among intra-regional coupling parameters, which are log scale parameters that ensure inhibitory responses, self-connectivity in the precuneus was significantly positively associated with SHS scores (*β* = 0.30, *t*(45) = 2.26, *p* < 0.05, *r* = 0.32; Fig. 3); however, this was not the case for self-connectivity in the amygdala (*β* = 0.09, *t*(45) = 0.60, *p* > 0.1, *r* = 0.09).

We conducted partial correlation analysis (controlling for sex, age, full-scale IQ, and FD effects) between these significant effective connectivity parameters and fALFF or functional connectivity data. A significant positive association was observed between the coupling parameters from the amygdala to the precuneus and functional connectivity between the amygdala and precuneus (*r* = 0.38, *p* < 0.05), and a significant negative association between self-connectivity in the precuneus and fALFF in the precuneus (*r* = −0.40, *p* < 0.05).

## Discussion

The analysis of fALFF values showed a negative association between SHS scores and resting-state neural activity in the precuneus. This finding is consistent with previous resting-state fMRI studies showing that mindfulness meditation^15^ and depression^19,20^, both of which are related to SHS scores, were negatively and positively associated with resting-state neural activity in the precuneus, respectively. However, these studies did not directly assess the relationship between SHS scores and resting state precuneus activity. To the best of our knowledge, this is the first study to show that higher SHS scores were associated with decreased resting-state neural activity in the precuneus.

Based on our results, the association between increased SHS scores and decreased precuneus activity can aid in understanding the information-processing mechanisms underlying subjective happiness. Several previous neuroimaging studies have shown some (not mutually exclusive) information-processing functions associated with activity in the precuneus. First, the results from functional neuroimaging studies showed that precuneus activity was associated with self-referential mental activity^7,37–40^. Specifically, analytical, negative, and avoidance-related self-relevant thoughts were shown to be related to precuneus activation^37,38^. Second, several neuroimaging studies showed that precuneus activity was positively associated with the degree of mind-wandering, including stimulus-independent thoughts about the past and future^41–43^. Third, in a study on subjective experience during real-time feedback, activation of the posterior cingulate gyrus, which is adjacent to and densely connected with the precuneus^25^, was associated with clinging, attached experiences, such as ruminative thinking and craving^44,45^. Interestingly, previous psychological studies reported that all of these mental functions associated with precuneus activity were related to subjective happiness. In several studies, happy individuals were found to be less inclined to self-reflect than were unhappy individuals^46,47^. Conversely, other studies indicated that people with psychiatric disorders, who have low levels of subjective happiness^48–50^, had highly self-reflective mental activity^51–53^. Moreover, a recent study found that people who engaged in less mind wandering were happier^54^. Finally, in another study, less clinging individuals reported a higher degree of subjective wellbeing^55^. Together with these neural and psychological data, our results suggest that decreased resting-state activity in the precuneus, which is associated with reduced self-referentiality, mind-wandering, and/or clinging characteristics, could be associated with greater happiness.

Additionally, our DCM analyses showed that self-inhibition in the precuneus was positively associated with SHS scores. These results are consistent with previous findings showing that self-inhibition in the medial parietal region was weakened in smokers^56^, who generally obtain reduced SHS scores^57^. Our results also showed that self-inhibition in the precuneus was related to the fALFF value in this region. Because the self-inhibition identified in the DCM analysis is thought to be produced in the populations of superficial pyramidal cells and interneurons utilizing specific types of neurotransmitters, including gamma-aminobutyric acid^58,59^, the results may provide micro-level mechanistic explanations regarding the relationships between resting-state neural activity and subjective happiness.

Furthermore, our results revealed stronger functional connectivity between the amygdala and right precuneus and effective connectivity from the amygdala to the precuneus associated with SHS scores. These results are consistent with anatomical evidence showing the anatomical connection between the amygdala and medial parietal region^25,28^ and with neuroimaging findings showing reduced resting-state functional connectivity between the amygdala and precuneus in patients with depression^29–31^. Because substantial neuroscientific evidence indicates that the amygdala is involved in emotional processing^26,27^, this result is also in agreement with the psychological model proposing that subjective happiness consists of multiple components, including emotional information^2^.

The functional and effective connectivity from the amygdala to the precuneus associated with SHS scores may indicate the information-processing mechanisms underlying subjective happiness. Reportedly, positive functional connectivity implies integrated functioning among brain regions^24^, and effective connectivity indicates the directional information flow between regions^32^. Therefore, the findings indicate that emotional information processed in the amygdala needs to be forwarded and integrated into the computations (likely including processes such as the self-referential activity described above) in the precuneus to implement subjective happiness. Consistent with this idea, several psychological researchers have proposed that the beneficial effects of mindfulness meditation on psychological disorders are mediated by exposure to emotional processing^12,60,61^; a mindful attitude that is accepting of one’s own emotional processing reduces subjective ill-being. Several experimental psychological studies also showed that coordinated emotional processing is associated with subjective happiness^62^. For example, people whose positive emotions fluctuate less^63^, those who want to feel emotions that match the environment, including negative ones^64^, and those who have more coherent behavioral and experiential emotional responses^65^ tend to have higher levels of subjective happiness or other types of subjective wellbeing. Based on these data, we speculate that functional coupling between the amygdala and precuneus may provide the emotional processing input for subjective happiness.

Our findings may elucidate the neuroscientific basis for several psychological interventions aimed at increasing subjective happiness. Previous behavioral studies have found that subjective happiness can be increased through mindfulness meditation and increased engagement in tasks that require attention. Interestingly, several previous fMRI studies showed that mindfulness meditation^15^ and tasks demanding attention^66–68^ decreased resting-state activity in the precuneus. Based on these data, we speculate that the increased happiness associated with these techniques may relate to the reduced resting-state activity in the precuneus.

This study has several limitations. First, although a negative association was shown between SHS scores and precuneus activity, our relatively small sample size was insufficient to draw any conclusions regarding the null findings in other brain regions. In addition, several previous resting-state neuroimaging studies showed that the precuneus was functionally connected with several other brain regions and acts as the hub in a large-scale neural network termed the default mode network^7,69,70^. It may be possible that the amygdala–precuneus network investigated in our DCM is a sub-component of a more widespread network. Future studies with a larger sample size may identify spontaneous neural activity in other regions and connectivity among widespread neural networks associated with subjective happiness.

Second, we used only one measure of subjective happiness, the SHS. Although the SHS has several advantages, such as high reliability and validity, as described in the Introduction^5^, this scale may not address all aspects of happiness. For example, because it assesses the general and non-time-specific sense of happiness^2^, different measurements of happiness at particular periods of time may yield different ratings and identify different neural correlates. Because the SHS assesses global subjective happiness using four items, previous studies using other measures that more thoroughly investigated the emotional or cognitive aspects of subjective happiness produced positively correlated but not perfectly matched values^2,5^. This may also account for the discrepancies between our findings and those of a recent resting-state fMRI study using a different scale to test the cognitive component of subjective happiness, which showed associations involving different brain regions^71^. Additionally, relationships between the neural activity and connectivity underpinning SHS scores and those underpinning other related psychological constructs, such as depressive symptoms, remain unclear and worthy of investigation. Future studies using different scales are needed to deepen our understanding of the neural mechanisms underlying subjective happiness.

In conclusion, our investigation using questionnaires and resting-state fMRI data revealed a negative relationship between SHS scores and spontaneous resting-state neural activity in the right precuneus. Furthermore, functional and effective connectivity from the amygdala to the precuneus were associated with SHS scores. Our findings, together with evidence from previous studies regarding the information-processing functions of these brain regions, suggest the possibility that subjective happiness is associated with a reduction in wandering, clinging, and self-referential mental states, which are well integrated with emotional processing.

## Methods

### Participants

The present study included 51 volunteers (26 females; mean ± *SD* age, 22.5 ± 4.5 years). The participants were administered the Mini-International Neuropsychiatric Interview^72^, a short structured diagnostic interview, by a psychiatrist or psychologist. The interview revealed no neuropsychiatric conditions among participants. All participants were right-handed, as assessed by the Edinburgh Handedness Inventory^73^. After a detailed explanation of the experimental procedure, all participants provided informed consent. This study was approved by the Ethics Committee of the Primate Research Institute, Kyoto University, and was conducted in accordance with institutional ethical provisions and the Declaration of Helsinki.

### Psychological questionnaires

The Japanese version of the SHS^2,74^, a four-item measure of global subjective happiness, was used to measure the participants’ subjective happiness. A number of previous studies used the SHS and demonstrated its good-to-excellent reliability and validity across diverse populations; for example, the SHS showed high internal consistency (*α* = 0.85–0.95 in seven studies), high test–retest stability (*r* = 0.90 for 4 weeks), and high correlations with other theoretically related constructs, such as optimism (*r* = 0.47–0.62 in four studies)^5^. The present study was part of a larger project investigating personality and mental health.

### Procedure

The participants completed a resting-state task lasting 5 min. A small white fixation cross on a black background was continuously presented at the center of the screen. The participants were instructed to keep their eyes open, fixate on the cross, and relax without thinking about any specific contents.

### MRI acquisition

Image scanning was performed on a 3-T scanning system (MAGNETOM Trio, A Tim System; Siemens, Malvern, PA, USA) at the ATR Brain Activity Imaging Center using a 12-channel head coil. Small elastic pads were placed on both sides of the head to minimize head motion. The functional images consisted of 39 consecutive slices parallel to the plane of the anterior–posterior commissure, and were acquired in ascending order, covering the whole brain. A T2*-weighted gradient-echo echo-planar imaging sequence was used with the following parameters: repetition time = 2,500 ms; echo time = 30 ms; flip angle = 80°; matrix size = 64 × 64; voxel size = 3 × 3 × 4 mm. After the acquisition of functional images, a T1-weighted high-resolution anatomical image was obtained using a magnetization-prepared rapid-acquisition gradient-echo sequence (repetition time = 2,250 ms; echo time = 3.06 ms; flip angle = 9°; inversion time = 1,000; GRAPPA acceleration factor = 2; 208 sagittal slices; slice thickness = 1 mm; field of view = 256 × 256 mm; voxel size = 1 × 1 × 1 mm).

### Image analysis

Image analyses were performed using the Statistical Parametric Mapping (SPM) 12 package (http://www.fil.ion.ucl.ac.uk/spm) and the Data Processing Assistant for Resting-State fMRI (DPARSF) V4.0^75^ within the Data Processing & Analysis for Brain Imaging (DPABI) V2.0 (http://rfmri.org/dpabi)^76^ implemented in MATLAB R2015b (MathWorks, Natick, MA, USA).

The major steps involved in our image analysis were preprocessing, calculation of resting-state fMRI measures, statistical analysis, and DCM. For the preprocessing, the first five volumes were discarded to account for magnetization equilibrium effects, and the remaining 120 volumes were preprocessed. Functional images were corrected for slice timing differences and head movements. Data from all participants exhibited only small motion corrections (<2 mm). Since small amounts of head movement between volumes can produce systematic artifacts in resting-state fMRI measures, we further evaluated FD, which was defined as the sum of the absolute derivative values of the six realignment parameters^35^. The maximum FD was <0.5 mm with a mean ± *SD* across all participants of 0.11 ± 0.04. The FD values were later used in creating nuisance regressors and confounding covariates in the individual and group level analyses, respectively. Next, nuisance covariate regression in native space was conducted before the spatial normalization to minimize the effects of noise caused by cardiac and respiratory cycles, scanner drifts, and head movements. Nuisance regressors of six realignment parameters (three translations and three rotations) and six realignment parameters at one time point before, the 12 corresponding squared items (i.e., Friston 24-parameter model^77^), the linear trend, the white matter and cerebrospinal fluid signals, the mean global signal, and a constant term were included in the general linear model were regressed out from the fMRI time series. For the functional connectivity analysis, spike regression, which was shown to be effective in censoring motion artifacts^78^, was used to correct motion-contaminated volumes identified using a threshold of FD > 0.5 mm as well as their one neighbor back and two neighbors forward. Each contaminated volume was modeled as a separate nuisance regressor having a value of 1 at the contaminated time point and 0 elsewhere in the general linear model. The combination of these nuisance regressors showed good performance in removing motion-related artefacts^79^. The T1 anatomical- and noise-corrected functional images were normalized to Montreal Neurological Institute space using the anatomical image-based unified segmentation–spatial normalization approach^80^ after the T1 anatomical image was coregistered to the mean of the functional images. Finally, the spatially normalized noise-corrected functional images were resampled to a voxel size of 3 × 3 × 3 and smoothed with an isotopic Gaussian kernel of 4-mm full-width at half-maximum, which is recommended for analyses of fALFF and functional connectivity.

To measure regional intrinsic brain activities in the resting state, fALFF was computed using the individual preprocessed data^10,11^. fALFF is the ratio between the sum of Fourier amplitudes within a specific low-frequency range (0.01–0.1 Hz) and the sum of Fourier amplitudes across the entire frequency range (0–0.2 Hz). The fALFF calculation was repeated for each voxel in the whole brain to create a fALFF map for each participant that was entered into the group level analysis. The fALFF value at each voxel was standardized by the global mean of the fALFF map to reduce the potential variability of global effects across participants.

To measure functional connectivity between the amygdala and the rest of the brain, seed-based functional connectivity analysis was conducted. First, the individual preprocessed data were bandpass-filtered at 0.01–0.1 Hz. The bilateral amygdala ROIs were obtained from the Automated Anatomical Labeling (AAL) atlas^81^. The fMRI time series data were extracted from each amygdala seed in the filtered data, and then Pearson’s correlation coefficients were calculated between the amygdala time series and the time series of all other voxels in the brain. The correlation coefficient at each voxel was transformed to a *z*-value using Fisher’s *r*-to-*z* transformation to improve normality. The resultant amygdala functional connectivity map for each participant was entered into the group level analysis.

To identify significant associations between SHS scores and regional intrinsic brain activities or the functional connectivity of the bilateral amygdala seed region, a voxel-wise multiple regression analysis was conducted using the fALFF value or the amygdala functional connectivity as the dependent variable, the SHS score as the independent variable, and sex, age, and full-scale IQ as covariates of no interest. To control head movements in the group level analysis, the mean FD, averaged across the entire time series, was also added as a covariate to minimize the impact of motion-related variance on group inference^78,82^. The relationship between SHS scores and fALFF values or the amygdala functional connectivity was tested using *t*-statistics and reported as a *z*-score after the *t*-value was transformed into the standard normal distribution. Clusters were considered statistically significant if they reached the extent threshold of *p* < 0.05, FWE-corrected for the whole brain, with a cluster-forming threshold (CFT) of *p* < 0.01 (uncorrected). Based on a recent study^83^, the parametric cluster size inference may inflate the false positive rate at a CFT of *p* < 0.01. Therefore, to validate our results, follow-up analyses were conducted using Permutation Analysis of Linear Models (PALM) software^84^, which is a permutation-based inference tool for nonparametric statistics. The extent threshold for significance was set at *p* < 0.05 FWE-corrected at a CFT of *p* < 0.01. Only the effects that reached statistical significance in both parametric and nonparametric analyses were reported. To scrutinize the results of the voxel-wise multiple regression analysis, ROI-based partial correlation coefficients between the fALFF value at the peak voxel or the first eigenvariate of fALFF values over the cluster and the SHS score were also calculated after controlling for the effects of sex, age, full-scale IQ, and FD.

The brain structures were labeled anatomically using the Talairach Client (http://www.talairach.org/)^85^ and AAL atlas^81^ included in the MRIcron software (www.mccauslandcenter.sc.edu/crnl/mricron/).

The relationships between SHS scores and fALFF or amygdala functional connectivity values were illustrated by plotting the values extracted at the peak voxels against the SHS scores after adjusting for the effects of no interest by regressing out sex-, age-, full-scale IQ-, and mean FD-related variances.

To explore the effective connectivity between the amygdala and precuneus based on the above functional connectivity analyses, spDCM^33,34^ was conducted using DCM12 in the SPM12. SpDCM is an extension of DCM^32^ and analyzes the frequency domain of the fMRI data using the complex cross-spectral density among multi-region fMRI time series. This analysis allows for modeling of the neural fluctuations and effective connectivity^33^.

Based on the results from the fALFF and functional connectivity analyses and our focus described in the Introduction, the association between SHS scores and the effective connectivity between the amygdala and precuneus in the right hemisphere was investigated.

First, the precuneus ROI was defined as the voxels belonging to the significant cluster (cluster size = 70 voxels, peak coordinates: *x* = 3, *y* = −72, *z* = −36) in the fALFF analysis; the right amygdala ROI obtained from the AAL atlas was the same ROI used for the seed-based functional connectivity analysis above. Next, the ROI time series were extracted from the DPARSF/DPABI preprocessed data for each participant as the first eigenvariate of all voxels within each ROI, and then converted into the cross spectra using a fourth-order autoregressive model with the frequency of interest ranging from 0.01 to 0.1 Hz.

Next, four hypothesized network models were constructed for each participant. The amygdala and precuneus were assumed to have either bidirectional, unidirectional (from the amygdala to the precuneus or from the precuneus to the amygdala), or no extrinsic (between-region) connections. The amygdala and precuneus were assumed to have self- or recurrent connections by default. To examine the optimal network structure, we conducted a random-effects BMS^36^. We used exceedance probabilities as the evaluation measures based on the belief that a particular model was likely to be more accurate than any other model given the group data. Based on the results of the random-effects BMS, we further analyzed the model with bidirectional connectivity between the amygdala and precuneus. The endogenous fluctuations were parameterized by the power law with the amplitudes and exponents of the spectral density. The parameters of the endogenous fluctuations, the effective connectivity, and the regionally specific hemodynamics were estimated to fit the observed cross spectra using the variational Bayesian techniques, a standard estimation scheme of DCM^86^.

To identify significant associations between SHS scores and effective connectivity, multiple regression analyses were performed using the coupling parameters for the extrinsic connections as the dependent variable, SHS scores as the independent variable, and sex, age, full-scale IQ and the mean FD as covariates of no interest. ROI-based partial correlation coefficients between the coupling parameters and SHS scores were also calculated controlling for the effects of sex, age, full -scale IQ, and FD. In addition, we conducted an exploratory partial correlation analysis between the significant effective connectivity parameters and fALFF or functional connectivity data controlling for the effects of sex, age, full-scale IQ, and FD.

## Supplementary information

Supplementary Figures 1 & 2
