# Supplementary material for: Resting-state neural activity and connectivity associated with subjective happiness
Source: Sci Rep. 2019 Aug 20;9:12098. doi: 10.1038/s41598-019-48510-9 (PMC6702218; doi:10.1038/s41598-019-48510-9)
Supplement: Supplementary file 1 — Supplementary Figures 1 & 2 [file 41598_2019_48510_MOESM1_ESM.doc]

Resting-state neural activity and connectivity associated with subjective happiness

Wataru Sato, Takanori Kochiyama, Shota Uono, Reiko Sawada, Yasutaka Kubota, Sayaka Yoshimura, and Motomi Toichi

Supplementary data


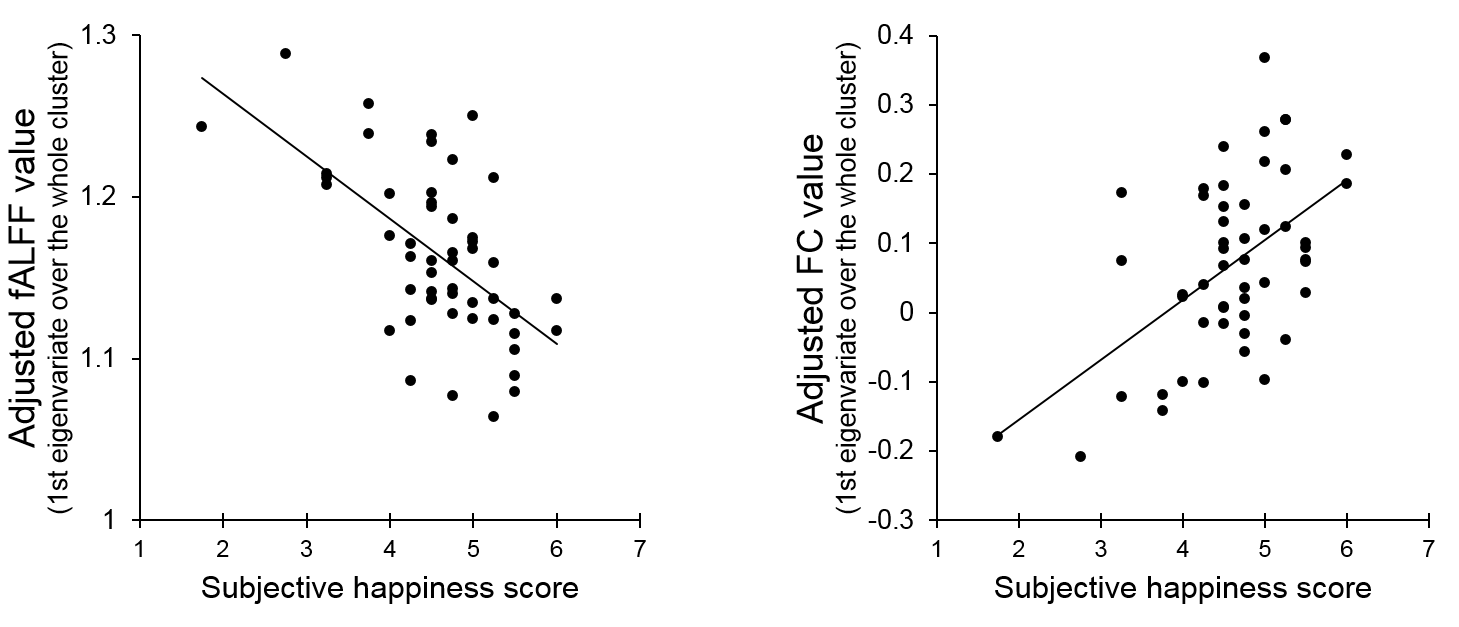


Supplementary Figure 1. Scatter plots of the first eigenvariate of the adjusted fractional amplitude of low-frequency fluctuation (fALFF) values (left) and functoional connectivity (FC) parameters (right) over the whole precuneous cluster as a function of the subjective happiness score. Effects of no interest (age, sex, full-scale intelligence quotient, and mean framewise displacement) were regressed out.


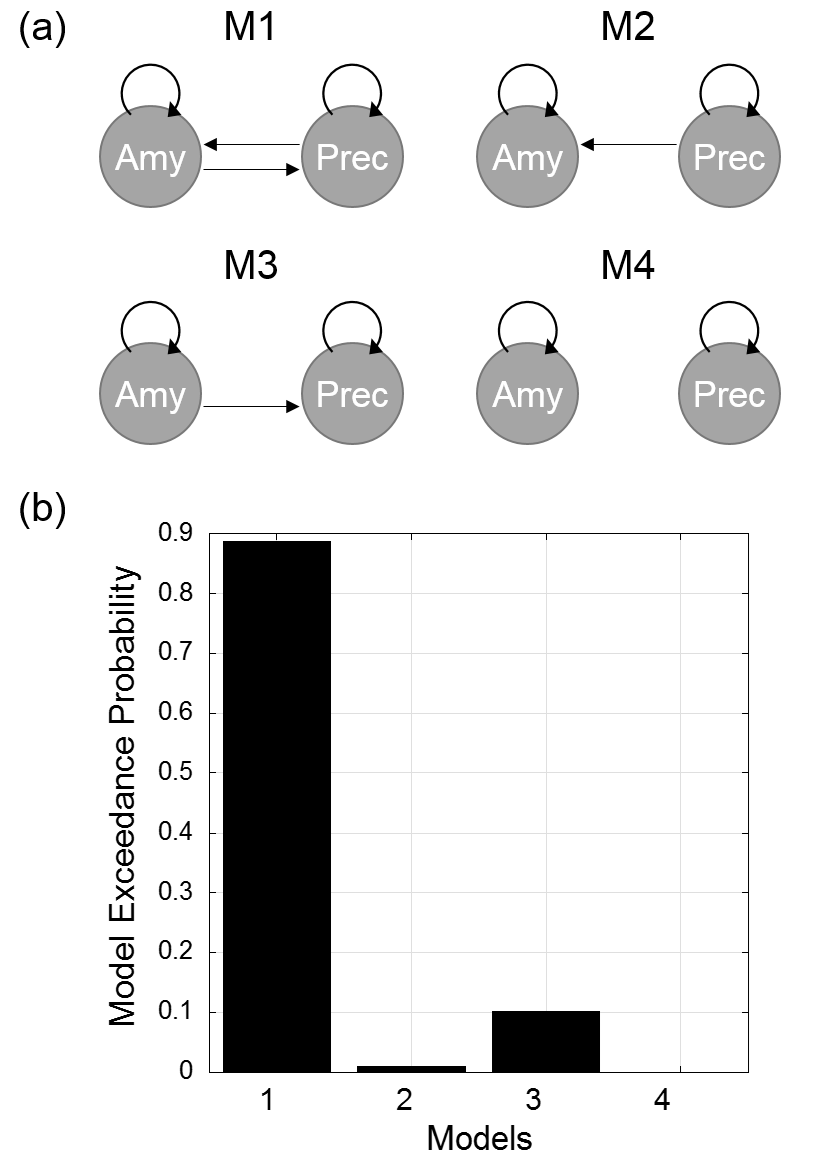


Supplementary Figure 2. Dynamic causal models we tested (a) and the exceedance probabilities of the models (b). The arrows indicate extrinsic (between-region) and inhibitory self (recurrent) connections in the amygdala (Amy) and precuneus (Prec).
